# The NGS Magic Pudding: A Nanopore-Led Long-Read Genome Assembly for the Commercial Australian Freshwater Crayfish, *Cherax destructor*

**DOI:** 10.3389/fgene.2021.695763

**Published:** 2022-01-19

**Authors:** Christopher M. Austin, Laurence J. Croft, Frederic Grandjean, Han Ming Gan

**Affiliations:** ^1^ Deakin Genomics Centre, Deakin University, Geelong, VIC, Australia; ^2^ Centre for Integrative Ecology, School of Life and Environmental Sciences, Deakin University, Geelong, VIC, Australia; ^3^ Laboratoire Ecologie et Biologie des Interactions, Equipe Ecologie Evolution Symbiose, Unité Mixte de Recherche 7267 Centre National de la Recherche Scientifique, Université de Poitiers, Poitiers, France; ^4^ GeneSEQ Sdn Bhd, Rawang, Malaysia

**Keywords:** genome, annotation, nanopore, cellulase, aquacultrure, decapoda, Parastacidae

## Abstract

*Cherax destructor*, the yabby, is an iconic Australian freshwater crayfish species, which, similar to other major invertebrate groups, is grossly under-represented in genomic databases. The yabby is also the principal commercial freshwater crustacean species in Australia subject to explotation via inland fisheries and aquaculture. To address the genomics knowledge gap for this species and explore cost effective and efficient methods for genome assembly, we generated 106.8 gb of Nanopore reads and performed a long-read only assembly of the *Cherax destructor* genome. On a mini-server configured with an ultra-fast swap space, the *de novo* assembly took 131 h (∼5.5 days). Genome polishing with 126.3 gb of PCR-Free Illumina reads generated an assembled genome size of 3.3 gb (74.6% BUSCO completeness) with a contig N_50_ of 80,900 bp, making it the most contiguous for freshwater crayfish genome assemblies. We found an unusually large number of cellulase genes within the yabby genome which is relevant to understanding the nutritional biology, commercial feed development, and ecological role of this species and crayfish more generally. These resources will be useful for genomic research on freshwater crayfish and our methods for rapid and super-efficient genome assembly will have wide application.

## 1 Introduction

Australia’s freshwater crayfish are highly diverse and as charismatic as the country’s better known avian and mammalian fauna, but far less appreciated and studied. Crayfish are found in a range of freshwater environments, include some exceptionally large species in Australia, and can reach very high densities in both natural and cultured environments (Nyström and Strand, 1996; Whitledge and Rabeni, 1997; Jones and Ruscoe, 2000; Reynolds and Richardson, 2013). As a result, they often represent keystone species and ecosystem engineers in permanent and semi-permanent freshwater systems in many parts of the world. This also means they are an important part of food webs as significant prey items for fish, birds and mammals (Hicks and McCaughan, 1997; Jones and Grey, 2016), and for humans, including indigenous communities (Eyre et al., 1845; Austin, 1998; Kusabs and Quinn, 2009). Crayfish also have significant ecological roles within inland aquatic systems as they can consume and process sizeable volumes of a range of organic matter and detritus (Nyström and Strand, 1996; Whitledge and Rabeni, 1997; Reynolds and Richardson, 2013; Jones and Grey, 2016). While crayfish are widely considered as omnivorous and opportunistic feeders their exact ecological role and nutritional biology has been controversial (Momot, 1995) and are assuming greater importance with the frequent translocation of crayfish species and their potential to cause a range of negative ecological impacts both locally and globally (Austin and Ryan, 2002; Lodge et al., 2012; James et al., 2016; Souty-Grosset et al., 2016). Some authors have postulated that freshwater crayfish are primarily carnivorous (Momot, 1995; Weinländer and Füreder, 2012), however molecular and limited NGS-based studied have revealed the presence of cellulase and a diversity of carbohydrate-active related genes supporting an adaption to the processing of plant-based food (Crawford et al., 2005; Tan et al., 2016). The first cellulase reported for freshwater crayfish was from the GH9 family which was found to be especially diverse in *Cherax quadricarinatus* based on a transcriptomic study by Tan et al. (2015) Tan et al. (2016).

**FIGURE 1:**
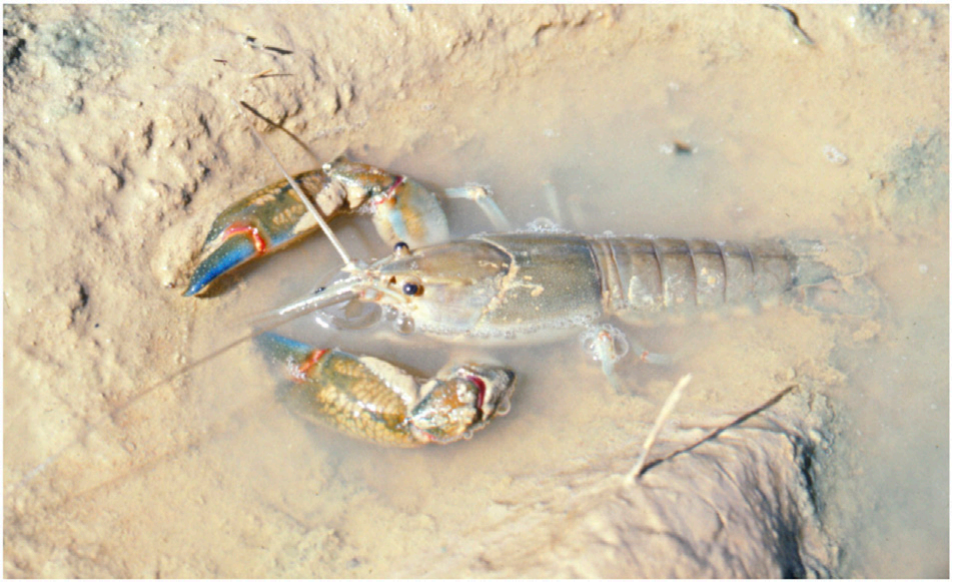
Adult *Cherax destructor*. Photo provided by Christopher Austin.

To date only one crayfish genome is available for the northern hemisphere species, *Procamabarus virginalis* (Cambaridae) and the southern hemisphere *Cherax quadricarinatus* (Parastacidae). *Cherax destructor*, commonly known as the yabby (Figure 1), is an iconic Australian freshwater crayfish species with a wide distribution throughout the river systems, lakes, swamps, and billabongs^1^ of inland Australia (Horwitz and Knott, 1995; Nguyen et al., 2004). It is the major commercial freshwater crayfish species in the country (Piper, 2000; Wingfield, 2008) and increasingly scientists are using it or closely related species as a model research species as they are easily maintained and bred in captivity (Mccarthy and Macmillan, 1999; Biro and Sampson, 2015; Beltz and Benton, 2017; Ventura et al., 2019). Despite the decreasing cost of whole-genome sequencing, publicly available whole-genome assemblies for freshwater crayfish species is scarce. Like many decapod crustaceans have large and repetitive genomes (Tan et al., 2020a) so short-read only *de novo* assemblies are memory-intensive and the resulting assemblies are often highly fragmented and difficult to annotate, thereby limiting their utility. While the supplementation of high coverage short-read data sets with low coverage (<10 ×) of long, but less accurate Nanopore or PacBio reads, is increasing the speed and quality of genome assemblies, it is still time-consuming, computationally demanding and challenging (Austin et al., 2017; Tan et al., 2018; Gan et al., 2019).

In this study, we sequence the genome of *Cherax destructor* and demonstrate that by starting with a medium coverage long read data set (∼20 × coverage) and similar coverage of Illumina reads for error-correction, the speed at which a quality reference genome can be produced can be greatly increased, even for species with large, and repetitive genomes. We benchmark our assembly against available genome assemblies for decapod crustaceans representing 11 species from a range of infraorders. Given the degree of ongoing interest in the nutritional biology and trophic status of freshwater crayfish, we also examine the diversity of cellulase genes in freshwater crayfish.

## 2 Methods

### 2.1 Genome Sequencing Libraries

A euthanized female crayfish specimen was provided by a local amateur angler in August 2019. The hepatopancreas tissue was dissected and homogenized in DNA/RNAshield (Zymo Research). Then, several gDNA extractions were performed on the homogenized hepatopancreas using the Zymo Quick gDNA kit (Zymo Research). For Nanopore sequencing, 20 µg of gDNA was fragmented to 8–10 kb using Covaris g-tube and 2–4 ug of the fragmented gDNA was subsequently used to construct a Nanopore library with the LSK109 library preparation kit. One-eighth of the eluted library volume was loaded onto an R9.4.1 revD flowcell followed by sequencing. Every 8–16 h, the run was stopped followed by a nuclease flush, library reload, and sequencing. Nanopore sequencing was performed on a total of 12 brand new and eight used (and nuclease flushed) flowcells. Base-calling of the fast5 reads used Guppy v3.3.3 (high accuracy mode). A total of 15,928,097 passed reads were generated totalling to 106.8 gigabases (Mean length: 6,705 bp, Median Length: 5,861 bp and Read Length N_50_: 8,843 bp, Longest read length: 182,535 bp). For Illumina sequencing, 1 µg of gDNA was fragmented to 350 bp and processed using the TruSeq DNA PCR-Free Kit (Illumina). Sequencing was done on a NovaSeq6000 using a run configuration of 2 × 150 bp. A total of 418,053,185 paired-end reads were generated totaling to 126.3 gigabases.

### 2.2 Genome Assembly

Whole-genome assembly was performed on an Ubuntu 18.04 mini-server equipped with AMD EPYC 7551P 32-core processor, 256 GB physical RAM, and 750 GB swap space created on a RAID 0 (Redundant Array of Independent Disks) partition comprising two 1 TB drives. Nanopore reads and intermediate assembly files were all stored on a separate RAID 0 partition comprising four 4 TB hard drives. De novo assembly of the Nanopore reads used wtdbg 2.5 (Ruan and Li, 2019) with the options “-t 60 -p 19 -AS 2 -s 0.05 -L 3000 -g 6G --edge-min 2 --rescue-low-cov-edges”. Using this configuration, the *de novo* assembly took 131 h (∼5.5 days) to complete with a maximum memory usage of 607 GD. After the wtdbg assembly, one round of polishing with long reads was performed using the wtdbg 2.5 internal polishing tool, wtpoa-cns. For genome polishing with Illumina reads, two rounds of polishing with Pilon v1.22 (Walker et al., 2014) were carried out. The raw paired-end reads were first adapter, quality and poly-G trimmed with fastp v0.20.0 (Chen et al., 2018). For each round of pilon-polishing, the trimmed reads were aligned to the genome using bwa-mem v 0.7.17-r1188 (Li, 2013) followed by correction of individual base errors (SNPs) and small indels using the options “--diploid –fix bases”. To overcome memory limitation in Pilon due to large genome size, the genome was split into 10 smaller fasta files, processed with Pilon separately and merged back into a single fasta file. Transcriptome-guided scaffolding of the polished contigs was performed with P_RNA_scaffolder v1 (Zhu et al., 2018) using publicly available transcriptome data (Ali et al., 2015). The genome completeness was assessed using BUSCO v5 (Waterhouse et al., 2017) with the Arthropoda ortholog dataset (Arthropod odb10). Statistics of the resulting assembly were generated using QUAST v5.0.2 (Gurevich et al., 2013) and are presented in Table 1. Illumina and Nanopore reads were mapped to the final assembly using bwa-mem (Li, 2013) and minimap2 v2.17 (Li, 2018), respectively. The BAM files were separately processed in Qualimap2 v2.2.1 (Okonechnikov et al., 2016) to generate additional statistics for the genome assembly based on read alignment.

**TABLE 1:** Genome assembly and annotation statistics.

Parameter	Details
Organism	Cherax destructor (Australian yabby)
Isolate	CDF2 (female, wild population)
Bioproject	PRJNA588861
Biosample	SAMN13258587
Whole-genome GenBank accession	WNWK00000000
Assembled scaffold/contig length	3,336,744,225 bp/3,336,542,896 bp
Scaffold N_50_ (number of sequences)	87,184 bp (98,662)
Contig N_50_ (number of sequences)	80,900 bp (100,635)
GC content	41.43%
BUSCO completeness	74.6% Single-copy, 1.1% Duplicated
Arthropoda odb9 (*n* = 1,006)	15.1% Fragmented, 9.1% Missing
Number of predicted protein-coding genes	45,673
Number of predicted proteins	47,377
With InterPro signature	21,102 (44.5%)
With gene ontology (GO) term	14,068 (29.7%)

### 2.3 Repeat Annotation and Protein-Coding Gene Prediction

Repetitive regions were identified using RepeatModeler v1.0.11 (Smit and Hubley, 2010). The *de novo* generated repeat library (Gan et al., 2020) was subsequently used to soft-mask the genome assembly with RepeatMaskerv4.0.7 (Tarailo-Graovac and Chen, 2009) with the options “-no_is –div 40 –xsmall”. Using this repeat annotation approach, 61.34% of the genome has been repeat-masked with long interspersed nuclear elements (LINEs) being the most common repeat annotated (31%). For protein-coding gene prediction, BRAKER v2.1.4 (Hoff et al., 2019) was chosen since it can incorporate both RNA-sequencing data and closely related proteins for gene prediction training. Publicly available *Cherax destructor* transcriptome datasets (Ali et al., 2015) were downloaded and aligned to the genome using STAR v2.7.1a (Dobin et al., 2013). To obtain closely related protein sequences, all publicly available *Cherax quadricarinatus* transcriptome data were downloaded from NCBI-SRA as of 2nd December 2019, individually assembled using rnaSPAdes v3.13.0 (Bushmanova et al., 2019) followed by redundancy removal of the concatenated transcripts using EvidentialGene v2013.03.11 (Gilbert, 2019). *Cherax quadricarinatus* translated open reading frames that are larger than 200 amino acid residues and labelled as “complete” e.g., with intact 5′ and 3′ ends, were selected as the protein input (Gan et al., 2020) for training in BRAKER2 using default settings. Using Orthofinder v2.3.8 (Emms and Kelly, 2018), the initial predicted proteins from BRAKER2 were used as the input for orthologous clustering with the available proteomes of the red claw crayfish (*C. quadricarinatus*) (Tan et al., 2020a), pacific white shrimp (*Litopenaeus vannamei*) (Zhang et al., 2019), black tiger prawn (*Penaeus monodon*) (Quyen et al., 2020), marbled crayfish (*Procambarus virginalis*) (Gutekunst et al., 2018), and amphipod (*Parhyale hawaiensis*) (Kao et al., 2016). Then, the predicted *C. destructor* proteins that formed orthologous clusters with at least one of the decapod species were used for subsequent annotation and analysis. Specific comparisons of peptide homology were made with several decpod crustaceans including the recently published clawed lobster genome (clawed lobsters are from the clade most closely related to the freshwater crayfish) (Polinski et al., 2021), the southern hemisphere crayfish (*Cherax quadricarinatus*) using NCBI’s *blastx* (evalue 1e^−10^). Putative protein functions were inferred using InterproScan v5.35-74.0 (Jones et al., 2014) with the options “—iprlookup –goterms --dp”. Identification of Carbohydrate-Active enzymes (CAZy) in the selected crustacean proteomes used dbCAN2 v2.0.0 (Zhang et al., 2018) and the identified GH9 cellulases were further extracted and their diversity explored by phylogenetic analysis. The GH9 cellulases were first aligned with MUSCLE v3.8.31 (Edgar, 2004) followed by trimming in trimal v1.9 (Capella-Gutiérrez et al., 2009) (“-automated1” option) and phylogenetic construction in IqTree v1.6.10 (Nguyen et al., 2014) (“-m TESTNEW –bb 1,000” options). The unrooted IQTree maximum likelihood tree was annotated and visualized in TreeFig v1.4.3 (Rambaut, 2009).

### 2.4 Data Availability

Raw sequencing libraries have been deposited in NCBI-SRA under the BioProjectPRJNA588861. The genome assembly has been deposited in GenBank under the accession number WNWK000000 (the version described in this paper is WNWK01000000). The wtdbg2.5 assembly log file, intermediate *C. destructor* genome assemblies, repeat annotation, CAZy annotation, protein-coding gene prediction (GTF format), predicted genes, and proteins have been deposited in the Zenodo repository (Gan et al., 2020). The *C*. *quadricarinatus* RNASpades transcriptome assemblies, QUAST-generated genome statistics for all Decapod genomes re-analyzed in this paper and their BUSCO calculations are also deposited at Zenodo (Gan et al., 2020).

## 3 Results and Discussion

An alignment rate of more than 99.5% was observed for both Illumina and Nanopore reads with the most frequently observed sequencing depth of 29× and 23×, respectively. Assuming the sequencing depth with the highest observed frequency represents the coverage of the single-copy genomic region, the genome size of *Cherax destructor* is estimated to be 4.36–4.64 gb (Total sequencing yield in gigabases divided by single-copy coverage). This is consistent with genome size estimates for the northern hemisphere crayfish *Procambarus virginalis* (∼3.5 gb) and *Cherax quadricarinatus* (∼5 gb) (Tan et al., 2020a) making Australian crayfish larger than all other crustaceans so far sequenced with the exception of the prawn *Exoplaemon carinicauda* (9.5 gb).

Using 106.8 gb and 126.3 gb of Nanopore and Illumina data, respectively, a 3.3 gb genome assembly was generated with an estimated BUSCO score of 89.7% in less than a week. The assembled genome size was ∼27.0% smaller than the genome size estimate. This is quite a common outcome for decapod genome assemblies due to sequencing bias and their repetitive genomes (Tan et al., 2020a; Polinski et al., 2021) and was reflected in the uneven distribution of read depths across scaffolds in our study. Over 3,000 scafolds have over 300x coverage, compared with an average read depth of 111x, consistent with the occurence of a significant proportion of repeat regions and potentially contributing to the discrepency between the assembled genome size and the genome size estimate.

The contig N_50_ of 80,900 bp is the longest to date among currently available for freshwater crayfish genome assemblies. Comparisons with the recently sequenced *Cherax quadricarinatus* genome (Tan et al., 2020a) initially assembled using short reads followed by scaffolding with low coverage Nanopore long reads, show that a long-read led assembly is more efficient though more costly. However, the higher cost of long reads is more than compensated for by increased computational efficiencies due to the availability of speedy and memory-efficient long-read assemblers (wtdbg2) (Ruan and Li, 2019) and lack of reliance on the need to generate large volumes of Illumina reads during the initial assembly stage.

**FIGURE 2:**
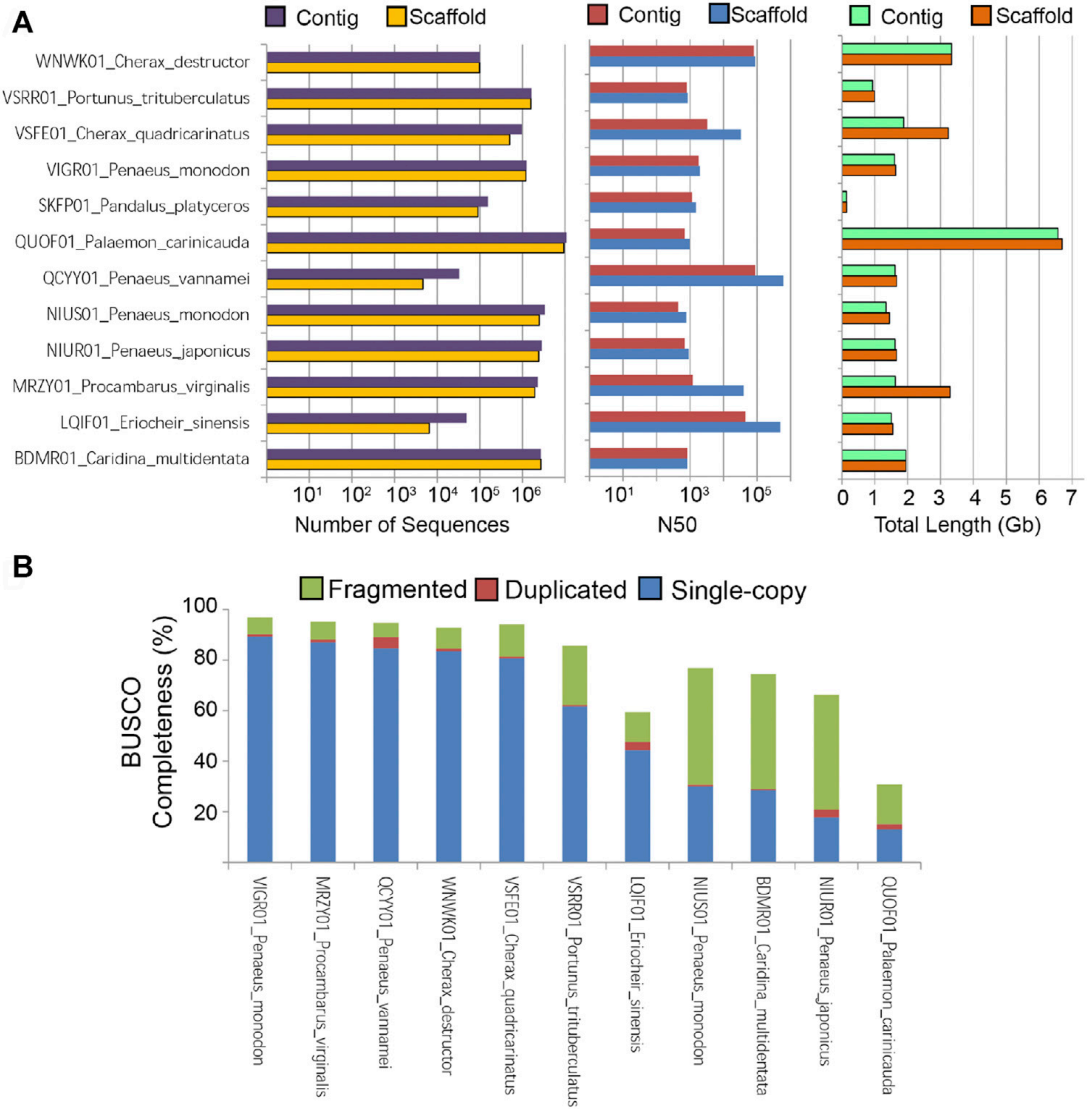
Statistics of publicly available decapod crustacean genome assemblies. **(A)** Number of sequences, Genome N_50_, and total assembled length **(B)** BUSCO completeness based on the Arthopoda ortholog dataset.

The cumulative scaffold length of *C. destructor* is similar to the *C. quadricarinatus* genome (∼3 gb) that was assembled using Illumina reads (191x) followed by scaffolding with low coverage Nanopore reads (x7). In comparison with the other two crayfish assemblies the advantages of a Nanopore-based assembly with an increased volume of long reads can be seen from Figure 2, where the difference between the contig and scaffold level assemblies is greatly reduced leading to a less gappy assembly. Also the need for high volumes of short reads is also greatly reduced with only 123.6 gb used in they study compared with used for the assemblies of *C. quadricarinatus* (964 gb) and *Procambarus virginalis* (350 gb).

It is also worth noting that this *C. destructor* genome assembly exhibits a contig N_50_ length of nearly 100 kb which is longest among freshwater crayfish genome assemblies. Recent decapod assemblies increasingly using both short and long reads and Hi-C data which is assisting in more robust decapod crustacean genome assemblies (Zhang et al., 2019; Tang et al., 2020) especiallyfor those species with large repetitive genomes such as *Macrobrachium* shrimps (Jin et al., 2021). The reported *C. destructor* BUSCO genome completeness in this study is also one of the highest to date for freshwater crayfish (Figure 2B). A logical next step, given the large and repetitve genomes exhinited by frewshwater crayfish, is to attempt to improve this genome assembly via the inclusion of HiC data (Jin et al., 2021).

**FIGURE 3:**
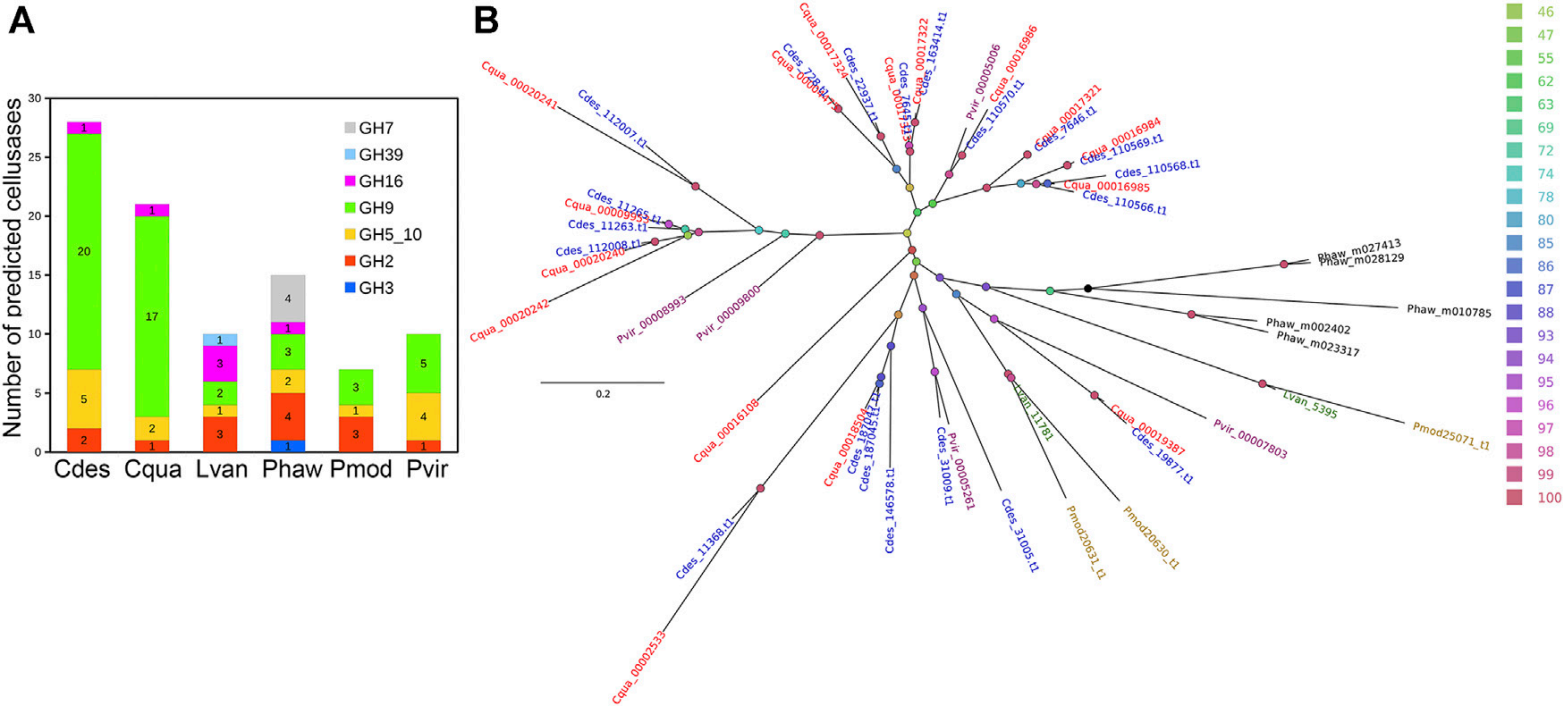
Identification and phylogenetic analysis of cellulases. **(A)** Number of identified cellulases in five decapod crustacean and an amphipod proteomes **(B)** IQTree maximum likelihood tree showing the evolutionary relationships of GH9 cellulases identified from the selected proteomes. The nodes were colored based on ultrafast bootstrap values and the first three letters in each tip label correspond to the species name. Branch lengths indicate number of substitutions per site. Cdes, *Cherax destructor*; Cqua, *Cherax quadricarinatus*; Lvan, *Litopenaeus vannamei*; Phaw, *Parhyale hawaiensis*; Pmod, *Penaeus monodon*; Pvir, *Procambarus virginalis*.

An initial 187,638 of putative unigenes were predicted by BRAKER2. The final protein set consisted of 47,377 transcripts (45,673 genes) of which 21,102 and 14,068 were identified with InterPro signature and Gene Ontology term, respectively. The number of predicted proteins with InterPro signatures is very similar to other species of decapod crustaceans. A total of 68.97% of *C. destructor* peptides mapped to the related *C. quadricarinatus* annotation (evalue 1e^−10^) (Tan et al., 2016). More specifically, we get 32,677 peptides in common with *Cherax quadricarinatus*, 25,129 with *Procambarus virginalis*, 23,008 with *Penaeus monodon*, 17,159 with *Litopenaeus vannamei*, and 10,318 with *Homarus americanus*. The number of predicted proteins with InterPro signatures is very similar to other species of decapod crustaceans (Tan et al., 2016). While the total number of predicted protein-coding genes is large (45,673) relative to those that have an Interpro signature, this number does not differ greatly from the recently published genome for the clawed lobster, *Hommarus americanus*, which identified 40,732 peptides (Polinski et al., 2021). This high proportion of unique genes is most likely a function of the evolution of a large repetitive genome and the limited genomic data for crayfish and lobsters as pointed out by Polinski et al. (2021) in their recent study of he American lobster (Polinski et al., 2021). Significantly*, Cherax destructor* harbours the highest number of cellulase genes among the currently sequenced decapod crustaceans (Figure 3A) with a substantially higher number of GH9 cellulase genes comparable to its close relative, *C. quadricarinatus*, which was previously highlighted in an earlier transcriptomic study (Tan et al., 2016). Phylogenetic analysis of the GH9 cellulases showed a clustering pattern first by the GH9 cellulase variants and then by species relatedness (Figure 3B). Despite the high number of GH9 cellulases identified among the *Cherax* spp., they were generally closely related and localized in a few major clades (Figure 3B). Although there were a few that claded with those from the northern hemisphere crayfish *P. virginalis*, indicating a more ancient origin. *Cherax destructor*, is considered to be versatile in its nutrient utilisation based on both dietary and field-based studies (Jones and De Silva, 1997; Beatty, 2006; Giling et al., 2009; Johnston et al., 2011) and is considered an opportunistic omnivorous generalist, that can derive nutrition directly from both animal and plant material and detritus.

A common view is that crayfish, in general, have a trophic role primarily as predators (Momot, 1995) may need to be re-assessed, given the antiquity, and diversity of cellulase and related genes in this group. However there also may be wide variation within and among crayfish species and the diet of particular species can vary in time and space (Beatty, 2006; Giling et al., 2009; Johnston et al., 2011) which has contributed to conflicting views. For example, Johnston et al. (2011) found variation between species from the same crayfish community ranging from primarily herbivorous species to primarily carnivorous species. Other species from this crayfish community, including *C. destructor*, had either mixed diets or switched between plant, and animal diets at different sites. It will, therefore, be of great interest to further examine cellulase diversity and expression in a range of crayfishes species from different environments including under aquaculture conditions and the ability of different crayfish species to utilise plant material in the field and through laboratory trials and how this relates to cellulase gene profiles and their expression.

In general, a significant limitation in further advancing the study of the genomics of non-model organisms is the computational resources and time needed to assemble genomes from predominately short reads, even when aided with long reads for scaffolding (Lewin et al., 2018). This problem is further exacerbated for groups with larger repetitive genomes, which means analyses can take months if not years and still lead to poor quality assemblies. In this study, we demonstrate that a high-quality genome assembly for a decapod crustacean with a large (>3 gb) and repetitive genome can be achieved with modest sequencing volumes, that take advantage of rapid and ongoing developments in third generation sequencing technologies, and can be completed in under 1 week of computation time on a high performance desktop machine.

## 4 Conclusion

This reference genome, along with its annotation, will be useful for future functional, ecological, aquaculture-related and evolutionary genomic studies, and genome-based selection and targeted genetic manipulation of this emerging aquaculture species. Given our finding of an evolutionary proliferation of cellulase genes, we are hoping these data will stimulate new research into the nutritional biology and trophic roles of freshwater crayfish in freshwater ecosystems. We see the continuing advances in Nanopore and other third generation sequencing technologies like the fabled “magic pudding” from a well known Australian children’s story (Norman, 1918), it keeps on “giving”, similar to the continuing inprovements in efficiency, output volume, and accuracy making the intractable, tractable when it comes to genome sequencing and assembly of non-model species. As a consequence we are able to provide a new model with respect to sequencing platforms, hardware configuration and assembly strategy to enable an ultrafast and efficient genome assembly that can be potentially applied to any species, including those with large and repetitive genomes. We anticipate our strategy and methodology will help elevate the study of interesting and important invertebrate genomes.

## Data Availability

The datasets presented in this study can be found in online repositories. The names of the repository/repositories and accession number(s) can be found below: https://www.ncbi.nlm.nih.gov/genbank/, PRJNA588861 https://www.ncbi.nlm.nih.gov/genbank/, SAMN13258587 https://www.ncbi.nlm.nih.gov/genbank/, WNWK00000000.1.

## Footnotes

1 Indigenous Australian name for a stagnant waterhole or river pool accepted into English.
